# Efficacy and Safety of Nefecon in Patients with IgA Nephropathy from Mainland China: 2-Year NefIgArd Trial Results

**DOI:** 10.34067/KID.0000000583

**Published:** 2024-10-09

**Authors:** Hong Zhang, Richard Lafayette, Bei Wang, Lisa Ying, Zhengying Zhu, Andrew Stone, Jens Kristensen, Jonathan Barratt

**Affiliations:** 1 Renal Division, Peking University First Hospital, Peking University Institute of Nephrology, Beijing, China; 2 Division of Nephrology, Department of Medicine, Stanford University, Stanford, California; 3 Everest Medicines Ltd., Shanghai, China; 4 Stone Biostatistics Ltd., Crewe, United Kingdom; 5 JDK Pharmaconsulting, Espergarde, Denmark; 6 The Mayer IgA Nephropathy Laboratories, Department of Cardiovascular Sciences, University of Leicester, Leicester, United Kingdom

**Keywords:** CKD, GFR, IgA nephropathy

## Abstract

**Key Points:**

- In the NefIgArd trial China cohort, 9 months of nefecon treatment led to clinically relevant preservation of kidney function over 2 years.
- Durable proteinuria reduction was also observed over 2 years with 9 months of nefecon treatment.
- These results were consistent with those of the global study population and support the disease-modifying effects of nefecon.

**Background:**

IgA nephropathy (IgAN), an immune-mediated kidney disease, is particularly prevalent among individuals of East Asian ancestry. Nefecon is a novel, oral, targeted-release budesonide formulation designed to inhibit galactose-deficient IgA1 formation underlying IgAN pathophysiology. We present findings in patients with IgAN from mainland China participating in the 2-year, multicenter, randomized, double-blind, phase 3 NefIgArd trial of nefecon.

**Methods:**

Patients (aged 18 years and older) with primary IgAN (eGFR 35–90 ml/min per 1.73 m^2^, persistent proteinuria [urine protein–creatinine ratio ≥0.8 g/g or proteinuria ≥1 g/24 hours] despite optimized renin-angiotensin system blockade) received nefecon or placebo over 9 months, followed by a 15-month follow-up phase on supportive care alone. The primary efficacy end point was time-weighted average of eGFR over 2 years.

**Results:**

Sixty-two patients from mainland China were included in this prespecified analysis. The primary efficacy end point was 9.6 ml/min per 1.73 m^2^ (95% confidence interval, 2.0 to 19.8) in favor of nefecon versus placebo. This was consistent with (and numerically greater than) that of the global study population. Time to confirmed 30% eGFR reduction or kidney failure from baseline was substantially delayed with nefecon (patients with an event: 9%) versus placebo (30%; hazard ratio, 0.21; 95% confidence interval, 0.04 to 0.73). No deaths were reported in the China cohort. In the nefecon group, treatment-emergent serious adverse events were reported by one patient during treatment and two patients during follow-up (versus no patients and seven patients, respectively, in the placebo group). No severe infections requiring hospitalization were reported.

**Conclusions:**

Nefecon treatment for 9 months showed greater preservation of eGFR over 2 years compared with placebo. The efficacy outcomes were consistent with global study results, with a numerically greater treatment benefit observed in patients from China. Nefecon was well tolerated, with no unexpected safety signals.

**Clinical Trial registry name and registration number:**

Efficacy and Safety of Nefecon in Patients With Primary IgAN, NCT03643965.

## Visual Abstract

**Figure:**
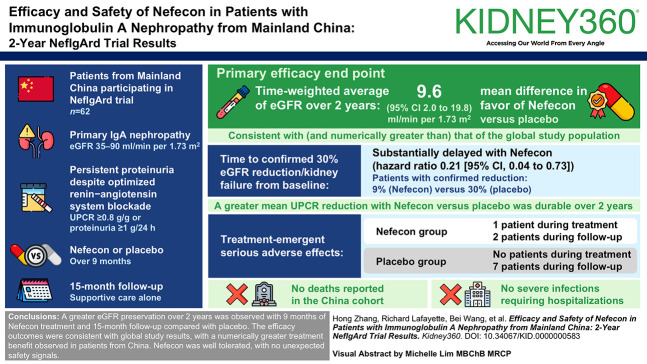

## Introduction

IgA nephropathy (IgAN) is a chronic immune-mediated kidney disease characterized by the deposition of immune complexes containing pathogenic galactose-deficient (Gd) IgA1 in the glomerular mesangium and is the most prevalent primary chronic glomerular disease worldwide.^1–4^ It is particularly prevalent among individuals of East Asian ancestry and exhibits a notably high incidence in China.^5–9^ In the Chinese population, IgAN accounts for 45%–58% of patients with GN, substantially higher than the 10%–35% observed in Europe.^6^ Furthermore, the male-to-female ratio in China for IgAN is approximately 1:1, in contrast to the European ratio of 3:1.^6^ With a population exceeding 1.4 billion, and given that a considerable proportion of patients with IgAN progress to kidney failure within approximately 20 years of presentation, the prevalence of IgAN in China also raises concerns about its effect on public health.^6,10^

Before the approval of nefecon (Calliditas Therapeutics) in China, there was no targeted treatment available for IgAN, with management consisting of optimized supportive care (including maximally tolerated renin-angiotensin system inhibition).^11,12^ At present, systemic corticosteroids are suggested for patients who remain at high risk of kidney disease progression despite optimized supportive care.^5^ However, their use should be carefully considered in clinical practice because of concerns over efficacy and adverse effects.^5,13,14^ In China, mycophenolate mofetil is often used as a corticosteroid-sparing agent, with its use becoming more widely accepted after reference to it in the Kidney Disease Improving Global Outcomes 2021 guidelines.^5,11,15–17^ However, this guidance is based on limited data availability.^5^ Similarly, the Kidney Disease Improving Global Outcomes 2021 guidelines suggest that hydroxychloroquine can be considered in Chinese patients who remain at high risk of progression despite optimized supportive care,^5^ although this is also based on limited evidence.^18^

There is evidence that the gut mucosal immune system is involved in IgAN pathogenesis, with gut-associated lymphoid tissue being a significant source of mucosal IgA.^4^ Peyer's patches (aggregations of lymphoid follicles within the intestinal mucosal layer) are a key induction site for mucosal B cells that ultimately produce excess Gd-IgA1 in IgAN.^3,4^

Nefecon is a novel, oral, targeted-release capsule formulation of budesonide and is designed to act in the gut-associated lymphoid tissue–rich distal ileum to reduce excess Gd-IgA1 production.^3,4^ Previously published efficacy and safety results from 364 patients in the 2-year Phase 3 Efficacy and Safety of Nefecon in Patients With Primary IgA Nephropathy (NeflgArd) trial (NCT03643965) in patients with primary IgAN showed that 9 months of treatment with nefecon (16 mg/d) led to a statistically significant (5.1 ml/min per 1.73 m^2^) benefit in the primary end point, time-weighted average eGFR over 2 years, with nefecon versus placebo (*P <* 0.001).^4^ A durable reduction in proteinuria was also recorded in the nefecon group, with a 41% reduction in time-averaged urine protein–creatinine ratio (UPCR) between 12 and 24 months versus placebo (*P <* 0.001).^4^ Here, we report the efficacy and safety of nefecon 16 mg/d in patients from mainland China who participated in the NefIgArd trial (China cohort) and assess the consistency of the treatment effect versus the global study population.

## Methods

Full methods and results of the global NeflgArd trial have been published previously.^4^

### Study Design and Participants

NefIgArd was a Phase 3, multicenter, randomized, double-blind, placebo-controlled trial. Patients with primary IgAN received nefecon 16 mg/d or a corresponding placebo for 9 months, followed by a 15-month observational follow-up period off the study drug and receiving standard of care only. Of 395 randomized patients, 366 patients (including 33 patients from China) were included in the global part of the study analysis, with an additional 29 patients enrolled in China after global recruitment had ended (to support regulatory approval in China). Therefore, a total of 62 patients were recruited from China and included in this prespecified analysis (Supplemental Figure 1).

As previously described,^4^ the key eligibility criteria were adults (aged 18 years and older) with biopsy-confirmed primary IgAN, persistent proteinuria (UPCR ≥0.8 g/g or proteinuria ≥1 g/24 hours in two consecutive measurements of the same parameter over ≥2 weeks) despite optimized supportive care, and eGFR of 35−90 ml/min per 1.73 m^2^ estimated with the CKD Epidemiology Collaboration 2009 formula. Patients with poorly controlled type 1 or 2 diabetes (hemoglobin A1c [HbA1c] >8% [>64 mmol/mol]), any secondary form of IgAN or any non-IgAN GN, poorly controlled BP (≥140/90 mm Hg), or a kidney transplant were excluded.

### Randomization and Masking

Patients were randomly assigned (1:1) to receive nefecon 16 mg/d or a matching placebo for 9 months. Randomization was stratified according to baseline proteinuria (<2 or ≥2 g/24 hours), baseline eGFR (<60 or ≥60 ml/min per 1.73 m^2^), and region (Asia-Pacific, Europe, North America, or South America).^4^

Throughout the 2-year trial, patients, investigators, and site staff involved in study procedures, patient evaluation, data entry, and laboratory data analysis remained blinded to treatment assignment. Key efficacy end points were calculated at a central laboratory by personnel masked to treatment assignment. Strict masking protocols were maintained until the completion of the trial.

### Outcomes

The primary efficacy end point for the initial 1-year analysis was defined as the UPCR (on the basis of 24-hour urine collection) at 9 months after the first dose of the study drug compared with baseline. Secondary efficacy end points included change in eGFR at 9 months and 12 months (*i.e*., 3 months off the study drug) compared with baseline and change in urine albumin–creatinine ratio (UACR) at 9 months compared with baseline.^19^

The primary efficacy end point for the full 2-year analysis was defined as the time-weighted average of eGFR over 2 years with eGFR calculated by a central laboratory at each time point (two separate measures were obtained at both baseline and 24 months). The primary efficacy end point was analyzed using robust regression, having multiply imputed any missing data first in three phases: imputation, analysis, and pooling. Sensitivity and supplemental analyses of the primary end point were also conducted as per the global population and are detailed in the Supplemental Statistical Methods. The analysis of the primary supportive end point of 2-year eGFR total slope is also detailed in the Supplemental Statistical Methods. Secondary efficacy end points included the ratio of eGFR, UPCR, and UACR averaged between 12 and 24 months versus baseline; time to 30% reduction from baseline in eGFR or kidney failure (defined as dialysis for ≥1 month, kidney transplantation, sustained [≥1 month] eGFR <15 ml/min per 1.73 m^2^, or kidney-related death)^4^; proportion of patients without microhematuria from months 12 to 24; proportion of patients receiving rescue treatment; and time from the first dose of the study drug until receiving rescue medication.

Safety outcomes for the 2-year analysis included treatment-emergent adverse events (TEAEs) and treatment-emergent serious adverse events (TESAEs), vital signs, body weight, clinical laboratory variables, and physical examination findings.^4^ Further details on the safety outcomes can be found in the 2-year global study publication.^4^

### Statistical Analyses

The methodology used for the statistical analysis for the China cohort was, in general, the same as used for the global analysis.^4^ No *P* values were computed for comparisons between nefecon and placebo specific to the China cohort because the study was not independently powered within this specified group. Because of the limited sample size in the China cohort, subgroup analyses were not conducted.

## Results

The 62 patients (*n*=32 on nefecon; *n*=30 on placebo) were randomized and dosed across 18 clinical sites; four patients (*n*=3 on nefecon; *n*=1 on placebo) discontinued treatment early (Supplemental Figure 1).

Most of the patients (*n*=27 [84%] on nefecon; *n*=28 [93%] on placebo) completed the 2-year trial, defined as those with at least one valid eGFR value within the 24-month visit window. More than 98% of patients in both groups took at least 80% of the expected number of capsules.

Demographic and baseline characteristics were generally balanced between both treatment groups (Table 1). Patients in the China cohort were younger (38 versus 43 years), had a lower body mass index (24 versus 28 kg/m^2^), and had a higher percentage of female patients (47% versus 34%) compared with the global study population.

**Table 1:** Demographic and baseline characteristics of the NefIgArd China cohort and global study population (full analysis set)

Characteristic	China Cohort^a^		Global Study Population	
	Nefecon 16 mg (*N*=32)	Placebo (*N*=30)	Nefecon 16 mg (*N*=182)	Placebo (*N*=182)
**Age, yr**				
Median (IQR)	38 (31–42)	39 (31–47)	43 (36–50)	42 (34–49)
**Age distribution, yr, *n* (%)**				
<45	27 (84.4)	20 (66.7)	98 (53.8)	104 (57.1)
≥45	5 (15.6)	10 (33.3)	84 (46.2)	78 (42.9)
**Sex, *n* (%)**				
Male	17 (53.1)	16 (53.3)	117 (64.3)	123 (67.6)
Female	15 (46.9)	14 (46.7)	65 (35.7)	59 (32.4)
**Race, *n* (%)**				
Asian	32 (100)	30 (100)	43 (23.6)	40 (22.0)
**Baseline BMI, kg/m** ^**2**^				
*n*	32	30	180	178
Median (IQR)	25 (23–26)	24 (21–27)	28 (25–32)	27 (24–30)
**Baseline BP, mm Hg, median (IQR)**				
Systolic	120 (116–125)	120 (111–125)	126 (121–132)	124 (117–130)
Diastolic	81 (73–85)	81 (77–86)	79 (76–84)	79 (74–84)
**Baseline UPCR, g/g**				
Median (IQR)	1.38 (0.84–1.94)	1.18 (0.92–1.55)	1.28 (0.90–1.76)	1.25 (0.88–1.74)
**Baseline proteinuria**				
Median (IQR), g/24 h	1.98 (1.49–2.77)	1.62 (1.30–2.43)	2.29 (1.61–3.14)	2.17 (1.53–3.39)
<2 g/24 h, *n* (%)	17 (53.1)	17 (56.7)	78 (42.9)	79 (43.4)
≥2 g/24 h, *n* (%)	15 (46.9)	13 (43.3)	104 (57.1)	103 (56.6)
**Baseline UACR, g/g**				
Median (IQR)	1.11 (0.66–1.60)	0.90 (0.66–1.22)	0.99 (0.68–1.40)	0.98 (0.66–1.42)
**Baseline total urine albumin, g/24 h**				
Median (IQR)	1.55 (1.17–2.41)	1.20 (0.97–2.20)	1.77 (1.24–2.49)	1.70 (1.12–2.54)
**Baseline eGFR (CKD-EPI)**				
Median (IQR), ml/min per 1.73 m^2^	65.1 (39.6–78.5)	61.5 (48.4–77.0)	56.1 (45.5–71.0)	55.1 (46.0–67.7)
<60 ml/min per 1.73 m^2^, *n* (%)	14 (43.8)	13 (43.3)	109 (59.9)	109 (59.9)
≥60 ml/min per 1.73 m^2^, *n* (%)	18 (56.3)	17 (56.7)	73 (40.1)	73 (40.1)
**Microhematuria at randomization, *n* (%)**				
Yes	25 (78.1)	26 (86.7)	123 (67.6)	127 (69.8)
No	7 (21.9)	4 (13.3)	59 (32.4)	55 (30.2)
**Time since IgAN biopsy diagnosis at informed consent**				
Median (IQR), yr	1.8 (0.4–3.8)	2.1 (0.7–4.1)	2.4 (0.6–6.9)	2.6 (0.6–6.5)
Use of systemic corticosteroids or immunosuppressants for IgAN and/or non-IgAN indications before randomization, *n* (%)	2 (6.3)	0 (0.0)	15 (8.2)	19 (10.4)
**Use of any RAS blockade (ACE inhibitors or ARBs) before randomization, *n* (%)**				
ACE inhibitor alone	3 (9.4)	3 (10.0)	81 (44.5)	69 (37.9)
ARB alone	26 (81.3)	25 (83.3)	90 (49.5)	102 (56.0)
Both ACE inhibitor and ARB	2 (6.3)	1 (3.3)	8 (4.4)	8 (4.4)
Missing/not recorded	1 (3.1)	1 (3.3)	3 (1.6)	3 (1.6)
**Level of RAS blockade as a percentage of maximum allowable dose^b^**				
<50%	5 (15.6)	4 (13.3)	39/180 (21.7)	34/179 (19.0)
≥50%	27 (84.4)	26 (86.7)	141/180 (78.3)	145/179 (81.0)
**Prediabetic or diabetic status at baseline, *n* (%)**				
Diabetic	3 (9.4)	0 (0.0)	16 (8.8)	8 (4.4)
Prediabetic^c^	10 (31.3)	10 (33.3)	71 (39.0)	50 (27.5)
Neither diabetic nor prediabetic	19 (59.4)	20 (66.7)	95 (52.2)	124 (68.1)

%=100×*n*/*N*.

Baseline was defined as the last measurement before the first dose of the study drug. Baseline for systolic and diastolic BP was defined as the arithmetic mean of all measurements before the first dose of the study drug. Baseline proteinuria, eGFR, and total urine albumin were calculated as the geometric mean of the two consecutive measurements before srandomization. ACE, angiotensin-converting enzyme; ARB, angiotensin receptor blocker; BMI, body mass index; CKD-EPI, CKDEpidemiology Collaboration; FBG, fasting blood glucose; HbA1c, hemoglobin A1c; IgAN, IgA nephropathy; IQR, interquartile range; NefIgArd, efficacy and safety of nefecon in patients with primary IgA nephropathy; RAS, renin-angiotensin system; UACR, urine albumin–creatinine ratio; UPCR, urine protein–creatinine ratio.

a Of the patients included in the China cohort, 33 patients from China were randomized during the global study, with a further 29 patients randomized after global recruitment had ended.

b For patients taking both ACE inhibitors and ARBs, the sum of the % of the maximum allowed dose for each was summarized. Patients who were not recorded as having received RAS blockade are included in the <50% category. The dose received was not recorded for some patients; these patients are not included in the summary. The denominator is the number of patients who had data on the RAS blockade maximum allowed dose.

c Prediabetic status was defined as baseline hemoglobin A1c ≥5.7% or fasting blood glucose ≥100 mg/dl.

### Efficacy Outcomes

There was a mean difference in the time-weighted average eGFR over 2 years of 9.6 ml/min per 1.73 m^2^ (95% confidence interval [CI], 2.0 to 19.8) in favor of nefecon (Supplemental Table 1). This difference was consistent with (and numerically greater than) that of the global study population, which reported a statistically significant difference of 5.1 ml/min per 1.73 m^2^ (95% CI, 3.2 to 7.4; *P <* 0.001) in favor of nefecon (Supplemental Table 1). Results from sensitivity and supplemental analyses were consistent with those of the primary analysis of the China cohort (Supplemental Table 1).

**Figure 1:**
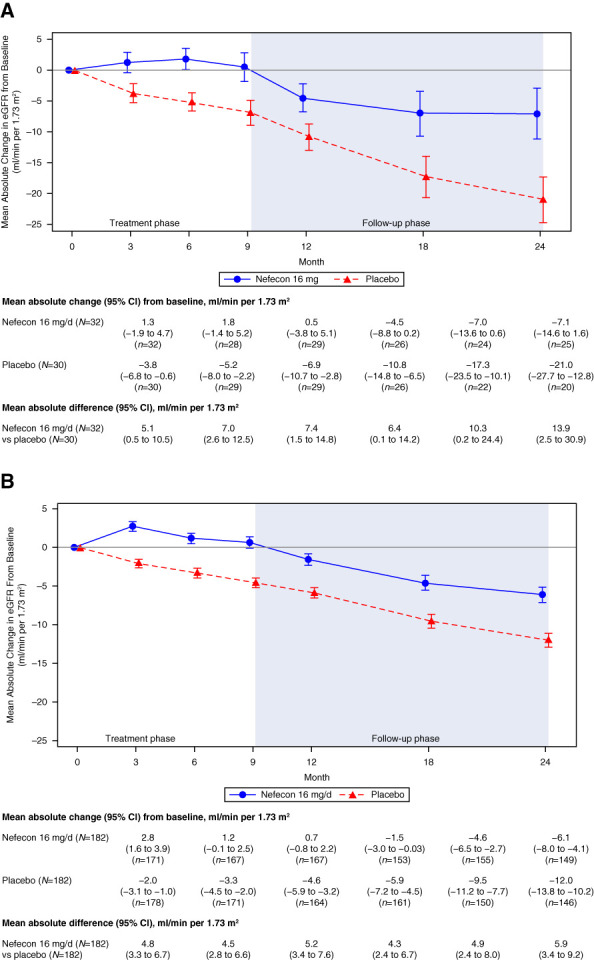
**Mean (±SEM) absolute change in eGFR from baseline in the NefIgArd trial.** (A) China cohort and (B) global study population (full analysis set). CI, confidence interval; NefIgArd, efficacy and safety of nefecon in patients with primary IgA nephropathy; SEM, standard error of the mean.

At 9 months, the mean absolute difference in eGFR between the nefecon and placebo groups was 7.4 ml/min per 1.73 m^2^ (95% CI, 1.5 to 14.8), which increased numerically over time to 13.9 ml/min per 1.73 m^2^ (95% CI, 2.5 to 30.9) at 24 months (Figure 1 and Supplemental Figure 2). This represented a preservation of 66% of kidney function in nefecon-treated patients compared with placebo-treated patients at 24 months and compared favorably with the global study population (Figure 1 and Supplemental Figure 2).^4^

In the primary supportive analysis of 2-year eGFR total slope, nefecon was associated with a clinically relevant benefit of 4.8 ml/min per 1.73 m^2^ (95% CI, 0.5 to 9.1) per year versus placebo (nefecon: −4.0 ml/min per 1.73 m^2^ per year [95% CI, −7.0 to −1.0]; versus placebo: −8.8 ml/min per 1.73 m^2^ per year [95% CI, −11.9 to −5.7]).

**Figure 2:**
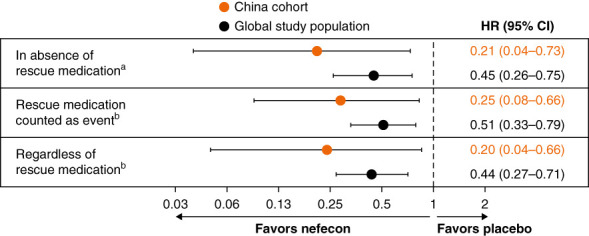
**Time to confirmed 30% eGFR reduction or kidney failure—NefIgArd China cohort and global study population.** Kidney failure was defined as dialysis for ≥1 month, kidney transplantation, sustained (≥1 month) eGFR <15 ml/min per 1.73 m^2^, or kidney-related death. HR, hazard ratio; IPCW, inverse probability of censoring weighting. ^a^The HR was estimated using an IPCW method. The aim of the analysis was to estimate the HR in the absence of rescue using IPCW because censoring because of rescue was considered informative. The 95% CIs were calculated using a profile-likelihood method. ^b^Standard Cox model, with 95% CIs calculated using a profile-likelihood method. HR, hazard ratio; IPCW, inverse probability of censoring weighting.

A treatment effect was also observed with nefecon versus placebo on the ratio of eGFR values averaged between 12 and 24 months versus baseline (Supplemental Table 2). Time to a confirmed 30% eGFR reduction from baseline or kidney failure event was substantially delayed in the nefecon versus placebo group (hazard ratio, 0.21; 95% CI, 0.04 to 0.73; 9% versus 30%, with a confirmed 30% eGFR reduction in the nefecon and placebo groups, respectively) (Figure 2). Results from the predefined supplemental analyses of the time to a confirmed 30% eGFR reduction or kidney failure event (with rescue medication included as an event or regardless of rescue medication) were consistent with the main analysis (Figure 2).

**Figure 3:**
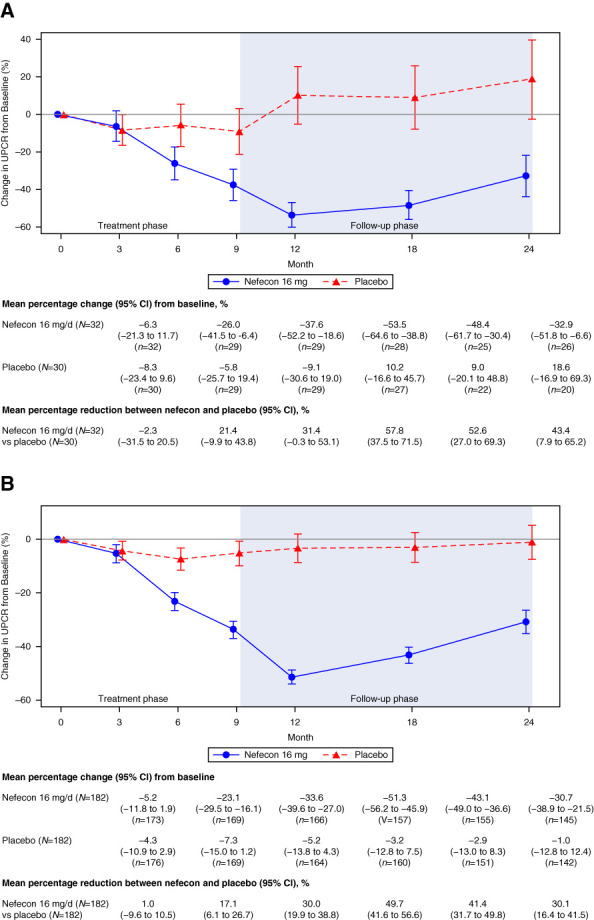
**Mean (±SEM) percentage change in UPCR from baseline in the NefIgArd trial.** (A) China cohort and (B) global study population (full analysis set). UPCR, urine protein–creatinine ratio.

A durable proteinuria reduction was observed in the China cohort, with a mean percentage reduction in UPCR from baseline that was 31% (95% CI, 0% to 53%) greater at 9 months and 43% (95% CI, 8% to 65%) greater at 24 months with nefecon versus placebo. These results were broadly in line with those of the global study population (Figure 3 and Supplemental Figure 3). A maximum reduction of 58% (95% CI, 38% to 72%) in the mean percentage change in UPCR from baseline was observed in the nefecon group versus placebo at 12 months. Analysis of the ratios of time-averaged UPCR and UACR over 12–24 months versus baseline identified reductions with nefecon of 52% (UPCR) and 53% (UACR) versus placebo (Supplemental Table 3).

**Figure 4:**
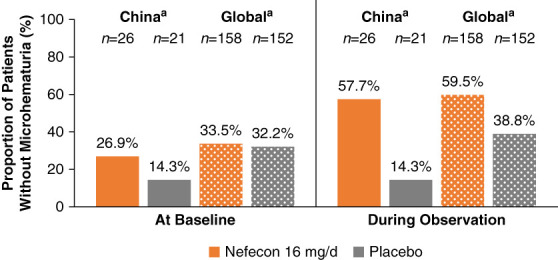
**Proportion of patients without microhematuria at baseline and during the observational period (12–24 months) in the NefIgArd China cohort and global study population.** ^a^*n* represents the number of patients with two or more valid urine dipstick results during the observational period.

There was a higher proportion of patients without microhematuria in the nefecon versus placebo group during the 12–24-month observational period (58% versus 14%, respectively); this compared favorably with the global study population (Figure 4). There was an imbalance in the proportion of patients without microhematuria at baseline between the nefecon and placebo groups of the China cohort; however, the positive result in favor of nefecon was confirmed by a *post hoc* sensitivity analysis that adjusted for the baseline imbalance (data not shown). Three patients in the nefecon group (9%) and ten patients in the placebo group (33%) had received rescue medication by 24 months (hazard ratio for time to receiving rescue medication, 0.22; 95% CI, 0.05 to 0.72; favoring nefecon) (Table 2).

**Table 2:** Analysis of time to rescue medication and proportion of patients receiving rescue treatment—NefIgArd China cohort and global study population (full analysis set)

Metric	China Cohort		Global Study Population	
	Nefecon 16 mg (*N*=32)	Placebo (*N*=30)	Nefecon 16 mg (*N*=182)	Placebo (*N*=182)
Received rescue medication in the first 9 mo, *n* (%)	1 (3.1)	2 (6.7)	7 (3.8)	5 (2.7)
Received rescue medication by 24 mo (*i.e*., at some point during the study), *n* (%)	3 (9.4)	10 (33.3)	15 (8.2)	20 (11.0)
**Comparison of time to rescue: nefecon 16 mg/d versus placebo^a^**				
HR (95% CI)^b^	0.22 (0.05 to 0.72)		0.68 (0.34 to 1.33)	

Rescue medication was defined as systemic immunosuppressive drugs (including glucocorticoids in some situations, see ref. 4), dialysis, and renal transplantation that could affect efficacy. A blinded medical review of concomitant therapy was performed before unblinding to confirm which patients had received rescue medication and at what time. Patients who received prohibited immunosuppressive medication for whom the dose and duration of treatment received were considered unlikely to have affected all subsequent efficacy data were not counted as an event for this analysis. CI, confidence interval; HR, hazard ratio; NefIgArd, efficacy and safety of nefecon in patients with primary IgA nephropathy.

a Comparison for the overall number of patients who received rescue medication at some point during the study.

b For the full analysis set of the China cohort, the HR and 95% CI (profile-likelihood method) are based on a Cox model with treatment, log-baseline urine protein–creatinine ratio, and log-baseline eGFR as covariates. For the global study population, the HR and 95% CI (profile-likelihood method) are based on a Cox model with treatment, log-baseline urine protein–creatinine ratio, log-baseline eGFR, and geographic region as covariates.

### Safety Outcomes

During the 9-month treatment period, TEAEs were experienced by 97% (*n*=31) of patients in the nefecon group and 80% (*n*=24) in the placebo group of the China cohort (Table 3). The most commonly reported TEAEs occurring in ≥15% of patients during treatment with nefecon are listed in Supplemental Table 4. One TEAE (albuminuria) required one patient in the nefecon group to discontinue treatment, but was considered mild in severity and unrelated to study treatment; no TEAEs were graded as severe. During the 15-month observational follow-up period, TEAEs were experienced by 72% (*n*=23) of patients in the nefecon group and 83% (*n*=25) in the placebo group, with most TEAEs being of mild or moderate severity. The most commonly reported TEAEs during follow-up with nefecon are listed in Supplemental Table 5. No deaths were reported.

**Table 3:** Overview of adverse events in the NefIgArd China cohort and global study population

Metric	China Cohort (Full Analysis Set)								Global Study Population (Full Analysis Set)							
	During 9-mo Treatment Period^a^				During 15-mo Follow-Up^b^				During 9-mo Treatment Period^a^				During 15-mo Follow-Up^b^			
	Nefecon 16 mg/d (*N*=32)		Placebo (*N*=30)		Nefecon 16 mg/d (*N*=32)		Placebo (*N*=30)		Nefecon 16 mg (*N*=182)		Placebo (*N*=182)		Nefecon 16 mg/d (*N*=182)		Placebo (*N*=182)	
	Pts, *n* (%)	e, *n*	Pts, *n* (%)	e, *n*	Pts, *n* (%)	e, *n*	Pts, *n* (%)	e, *n*	Pts, *n* (%)	e, *n*	Pts, *n* (%)	e, *n*	Pts, *n* (%)	e, *n*	Pts, *n* (%)	e, *n*
No. of Pts with a study visit during follow-up, *N*′	—		—		32		30		—		—		175		174	
Any TEAE	31 (96.9)	200	24 (80.0)	84	23 (71.9)	84	25 (83.3)	117	159 (87.4)	804	125 (68.7)	476	127 (72.6)	406	124 (71.3)	431^c^
**Maximum severity of TEAEs, *n* (%)**																
Mild	21 (65.6)	183	16 (53.3)	75	15 (46.9)	72	16 (53.3)	96	93 (51.1)	637	75 (41.2)	387	62 (35.4)	276	73 (42.0)	300
Moderate	10 (31.3)	17	8 (26.7)	9	7 (21.9)	11	7 (23.3)	17	57 (31.3)	155	46 (25.3)	84	49 (28.0)	110	43 (24.7)	115
Severe	0 (0.0)	0	0 (0.0)	0	1 (3.1)	1	2 (6.7)	4	9 (4.9)	12	4 (2.2)	5	16 (9.1)	20	8 (4.6)	15
Any TESAE	1 (3.1)	1	0 (0.0)	0	2 (6.3)	2	7 (23.3)	11	18 (9.9)	26	9 (4.9)	10	14 (8.0)	18	14 (8.0)	16
Any study treatment-related TESAE	1 (3.1)	1	0 (0.0)	0	1 (3.1)	1	0 (0.0)	0	4 (2.2)	4	4 (2.2)	5	0 (0.0)	0	1 (0.6)	2
Any AE leading to death	0 (0.0)	0	0 (0.0)	0	0 (0.0)	0	0 (0.0)	0	1 (0.5)	1	0 (0.0)	0	1 (0.6)	1	0 (0.0)	0
Any TEAE leading to discontinuation of study treatment	1 (3.1)	1	0 (0.0)	0	—	—	—	—	17 (9.3)	36	3 (1.6)	8	—	—	—	—

%=100×*n*/*N* (or 100×*n*/*N′*).

TEAEs were defined as adverse events that occurred for the first time after dosing with study treatment or existed before but worsened in severity or relationship to study treatment after dosing.

TESAEs were defined as serious adverse events that occurred for the first time after dosing with study treatment or existed before but worsened in severity or relationship to study treatment after dosing. AE, adverse event; e, events; NefIgArd, efficacy and safety of nefecon in patients with primary IgA nephropathy; Pts, patients; SAE, serious adverse event; TEAE, treatment-emergent adverse event; TESAE, treatment-emergent serious adverse event.

a Adverse events that started >14 days after the last dose of study treatment were excluded from the summary. The last dose was defined as the last dose the patient received, including the tapering period, regardless of the duration of treatment.

b An adverse event that occurred >14 days after the last dose of study treatment was not considered a treatment emergent adverse event if it was not the first occurrence, did not worsen in severity compared with the previous occurrence, and/or was not related to study treatment.

c One patient had a treatment-emergent adverse event of hematuria, the severity of which was not recorded (no reason for this omission was provided in the electronic case report form).

During the 9-month treatment period, TESAEs were reported by one patient (3%) in the nefecon group and none in the placebo group, whereas during the 15-month observational follow-up period, two patients (6%) in the nefecon group and seven patients (23%) in the placebo group experienced TESAEs (Supplemental Table 6).

During both the 9-month treatment period and 15-month follow-up period, no severe infections requiring hospitalization were reported in either treatment group.

Consistent with the global study population, there were no clinically relevant changes in median values of any chemistry parameters observed over time between groups in the China cohort. There were three nefecon-treated patients with an increase in HbA1c ≥1% at 9 months, all of whom had a pre-enrollment diagnosis of diabetes (Supplemental Figure 4). One of these patients had an increase in HbA1c ≥1% to <2.0%, whereas the other two had an increase in HbA1c ≥2%. The highest increase in HbA1c seen during nefecon treatment was 5.0%; this patient had diabetes and had discontinued empagliflozin treatment before enrollment (metformin treatment was started following a recorded HbA1c of 11.6%, and their HbA1c level subsequently returned to baseline). There were no clinically relevant trends for changes in vital signs observed over time in the China cohort, which included no clinically relevant treatment-related effects on BP (Supplemental Figure 5), and no relevant changes in body weight or body mass index from baseline or between treatment groups.

## Discussion

In the China cohort of the Phase 3 NeflgArd trial, 9 months of nefecon treatment provided clinically relevant preservation of eGFR and durable proteinuria reduction over 2 years versus placebo. The efficacy results compare favorably with those of the global study population, with the treatment effect of nefecon being numerically greater in the China cohort. It is important to note that there was a greater decline in eGFR in the placebo arm of the China cohort compared with the placebo arm in the global study population (Figure 1). However, it is generally accepted that patients of Asian origin are at an increased risk of rapid disease progression and eGFR decline compared with patients of other ancestries,^20^ which may be indicated by the steep eGFR decline with supportive care only in the placebo group of the China cohort (Figure 1). Several genome-wide association studies investigating IgAN susceptibility loci have identified genetic risk factors involved in IgA levels and mucosal immunity, with several risk alleles being of increased frequency in Asian versus European cohorts.^21,22^ The risk of patients experiencing the composite outcome of 30% eGFR reduction from baseline or kidney failure was also reduced in a clinically meaningful way in patients treated with nefecon in the China cohort compared with placebo-treated patients, providing evidence that nefecon positively affects the course of decline in kidney function in these patients from mainland China.

The proportion of patients in the China cohort without microhematuria during the observational follow-up was higher in those treated with nefecon versus placebo. Hematuria is an independent predictor of kidney disease progression, providing further evidence for the disease-targeting effect of nefecon on IgAN. From the directionally consistent (and numerically greater) efficacy results between the China cohort and global population, nefecon seems to be the first disease-specific treatment that can provide a long-term clinical benefit in Chinese patients with IgAN.

Nefecon was well tolerated in the China cohort, with a safety profile that was generally consistent with the global study and was as expected for an oral budesonide product, with no new clinically relevant safety findings. During observational follow-up, one nefecon-treated patient reported a serious adverse event (upper respiratory tract infection) that was considered by the investigator to be potentially related to treatment. However, this was because of local coronavirus disease 2019 regulations requiring the patient to be hospitalized because of intermittent fever. No cases of severe infection were recorded, in contrast with the Therapeutic Effects of Steroids in IgA Nephropathy Global and Supportive versus Immunosuppressive Therapy for the Treatment of Progressive IgA Nephropathy trial findings, in which significant rates of side effects were observed in patients with IgAN receiving systemic corticosteroids, including high rates of serious adverse events, excess hospitalizations, serious infections, and deaths.^13,23^ Together with the limited effects of nefecon on HbA1c and without observed differences in weight, these data suggest a low degree of systemic corticosteroid exposure with nefecon.

Given the relatively small size of the China cohort, formal statistical hypothesis testing or subgroup analyses were not planned. Despite this, however, the 95% CIs across the end points excluded no effect and, when compared with those of the statistically powered global study population, suggest that there are no specific regional or racial differences in the efficacy and safety of nefecon.

As mentioned previously, epidemiology data suggest that IgAN is most prevalent among individuals of East Asian ancestry and can progress more rapidly than in other populations.^6,20^ This highlights the need to initiate targeted, disease-modifying therapy early in these patients. Alongside the favorable efficacy and safety profile observed in the NefIgArd trial,^4^ biomarker analyses from the earlier Phase 2 trial, The Effect of Nefecon in Patients With Primary IgA Nephropathy at Risk of Developing End-stage Renal Disease, have shown that nefecon reduces levels of Gd-IgA1 and IgA-containing immune complexes versus placebo,^24^ lending further support to a disease-modifying action. Nefecon may be positioned as a suitable therapy for continuous suppression of Gd-IgA1 production, as opposed to a single 9-month treatment course as was conducted in this study. However, the safety and efficacy of additional courses of nefecon treatment have not been established; an open-label extension study with nefecon (nefecon NCT04541043) will help to address this. The present findings also call into question the continued use of treatments, such as mycophenolate mofetil or hydroxychloroquine, in clinical practice, for which the supportive clinical data are limited.^5^ These results, and potentially those of other targeted therapies in the future, should provide cause to reassess treatment practices in patients with IgAN in China.

Overall, these findings support a disease-modifying, targeted effect of nefecon in patients with primary IgAN from mainland China.

## Supplementary Material

## Data Availability

Partial restrictions to the data and/or materials apply. Data sharing requests will be considered on a case-by-case basis from research groups who submit a research proposal to Jens Kristensen (jens.kristensen@calliditas.com) with a valuable research question and appropriate statistical analysis and dissemination plans. Proposals will be assessed by a committee formed from the trial management group, including senior statistical and clinical representation. Deidentified data will be shared *via* a secure data access system.

## Supplemental Material

Supplemental Statistical Methods

Supplemental Table 1. Sensitivity and supplemental analyses of the time-weighted average of eGFR over 2 years—NefIgArd China cohort and global study population.

Supplemental Table 2. Ratio of eGFR averaged over 12–24 months compared with baseline using robust regression—NefIgArd China cohort and global study population (full analysis set).

Supplemental Table 3. Ratio of time-averaged UPCR or UACR between 12 and 24 months compared with baseline—NefIgArd China cohort and global study population.

Supplemental Table 4. TEAEs in the NefIgArd China cohort (≥15% in the nefecon treatment group) and global study population (full analysis set) during treatment.

Supplemental Table 5. TEAEs in the NefIgArd China cohort (≥5% in the nefecon treatment group) and global study population (full analysis set) during follow-up.

Supplemental Table 6. TESAEs in the NefIgArd China cohort during treatment and during follow-up.

Supplemental Figure 1. Patient disposition—NefIgArd China cohort (full analysis set).

Supplemental Figure 2. Mean absolute and percentage changes in eGFR at 9 and 24 months—NefIgArd China cohort and global study population.

Supplemental Figure 3. Mean percentage change in UPCR at 9 and 24 months—NefIgArd China cohort and global study population.

Supplemental Figure 4. HbA1c shift plots comparing months 3, 6, 9, 12, 18, and 24 with baseline—NefIgArd China cohort.

Supplemental Figure 5. Box plots of change from baseline in BP in the NefIgArd China cohort. (A) Systolic BP and (B) diastolic BP.
