# Supplementary material for: Efficacy and Safety of Nefecon in Patients with IgA Nephropathy from Mainland China: 2-Year NefIgArd Trial Results
Source: Kidney360. 2024 Oct 9;5(12):1881–92. doi: 10.34067/KID.0000000583 (PMC11687989; doi:10.34067/KID.0000000583)
Supplement: Supplementary file 1 [file kidney360-5-1881-s001.pdf]

ASN
American Society of Nephrology
JASN
Journal of the American Society of Nephrology
KIDNEY360
Accessing Our World From Every Angle
CJASN
Clinical Journal of the American Society of Nephrology

# ASN Journal Disclosure Form

As per ASN journal policy, I have disclosed any financial relationships or commitments I have held in the past 36 months as included below. I have listed my Current Employer below to indicate there is a relationship requiring disclosure. If no relationship exists, my Current Employer is not listed.

J. Barratt reports the following:
Employer: UNIVERSITY OF LEICESTER; Consultancy: Astellas, Alebund, Alnylam, Alpine, Argenx, BioCryst, Calliditas, Chinook, Dimerix, HiBio, Kira, Novartis, Omeros, Otsuka, Travere Therapeutics, Q32 Bio, Roche, Sanofi, Takeda, Vera Therapeutics, Vifor, Visterra; Research Funding: Novartis; GlaxoSmithKline; Calliditas, Visterra, Chinook, Omeros, Galapagos, argenx, Travere Therapeutics; and Advisory or Leadership Role: Editorial Board of Kidney International, CJASN, Glomerular Diseases & Clinical Science; Treasurer International IgA Nephropathy Network.

I understand that the information above will be published within the journal article, if accepted, and that failure to comply and/or to accurately and completely report the potential financial conflicts of interest could lead to the following: 1) Prior to publication, article rejection, or 2) Post-publication, sanctions ranging from, but not limited to, issuing a correction, reporting the inaccurate information to the authors' institution, banning authors from submitting work to ASN journals for varying lengths of time, and/or retraction of the published work.

Name: Jonathan Barratt
Manuscript ID: K360-2024-000443R1
Manuscript Title: Efficacy and Safety of Nefecon in Patients With Immunoglobulin A Nephropathy From Mainland China: 2-Year NefIgArd Trial Results
Date of Completion: August 9, 2024
Disclosure Updated Date: March 21, 2024

ASN American Society of Nephrology | JASN Journal of the American Society of Nephrology | KIDNEY360 Accessing Our World From Every Angle | CJASN Clinical Journal of the American Society of Nephrology

# ASN Journal Disclosure Form

As per ASN journal policy, I have disclosed any financial relationships or commitments I have held in the past 36 months as included below. I have listed my Current Employer below to indicate there is a relationship requiring disclosure. If no relationship exists, my Current Employer is not listed.

J. Kristensen reports the following:
Consultancy: Calliditas Therapeutics AB

I understand that the information above will be published within the journal article, if accepted, and that failure to comply and/or to accurately and completely report the potential financial conflicts of interest could lead to the following: 1) Prior to publication, article rejection, or 2) Post-publication, sanctions ranging from, but not limited to, issuing a correction, reporting the inaccurate information to the authors' institution, banning authors from submitting work to ASN journals for varying lengths of time, and/or retraction of the published work.

Name: Jens Kristensen
Manuscript ID: K360-2024-000443R1
Manuscript Title: Efficacy and Safety of Nefecon in Patients With Immunoglobulin A Nephropathy From Mainland China: 2-Year NefIgArd Trial Results
Date of Completion: August 14, 2024
Disclosure Updated Date: August 14, 2024

ASN American Society of Nephrology | JASN Journal of the American Society of Nephrology | KIDNEY360 Accessing Our World From Every Angle | CJASN Clinical Journal of the American Society of Nephrology

# ASN Journal Disclosure Form

As per ASN journal policy, I have disclosed any financial relationships or commitments I have held in the past 36 months as included below. I have listed my Current Employer below to indicate there is a relationship requiring disclosure. If no relationship exists, my Current Employer is not listed.

R. Lafayette reports the following:
Employer: Stanford University; Consultancy: Calliditas, Inc; Chinook, Inc: Omeros, Inc; Otsuka, Inc. ;  Alexion, Inc, Travere, Inc., Vera, inc. , Novartis, Aurinia; Visterra, Alpine Bio, Beigene.; and Research Funding: NIH, Pfizer, Roche, Amgen, Otsuka, Omeros, Calliditas, Travere, Apellis, Chinook, Vera, Beigene, .

I understand that the information above will be published within the journal article, if accepted, and that failure to comply and/or to accurately and completely report the potential financial conflicts of interest could lead to the following: 1) Prior to publication, article rejection, or 2) Post-publication, sanctions ranging from, but not limited to, issuing a correction, reporting the inaccurate information to the authors' institution, banning authors from submitting work to ASN journals for varying lengths of time, and/or retraction of the published work.

Name: Richard A. Lafayette
Manuscript ID: K360-2024-000443R1
Manuscript Title: Efficacy and Safety of Nefecon in Patients With Immunoglobulin A Nephropathy From Mainland China
Date of Completion: August 7, 2024
Disclosure Updated Date: May 1, 2024

ASN American Society of Nephrology | JASN Journal of the American Society of Nephrology | KIDNEY360 Accessing Our World From Every Angle | CJASN Clinical Journal of the American Society of Nephrology

# ASN Journal Disclosure Form

As per ASN journal policy, I have disclosed any financial relationships or commitments I have held in the past 36 months as included below. I have listed my Current Employer below to indicate there is a relationship requiring disclosure. If no relationship exists, my Current Employer is not listed.

A. Stone reports the following:
Employer: Stone Biostatistics Ltd; Consultancy: Accession, Adaptimmune, Adcendo,Akamis, AstraZeneca, Biosight; Calliditas; Cogent, Confo, Crossbow, Dizal; GSK; GreyWolf, Intellia, Keros, Lava, Merus, OneCarbon, Orna, Purespring, Repare, Synklino, UroGen, VarmX,; and Other Interests or Relationships: I serve, or have served on and IDMC in the last 24 months for the following companies. I wish to keep these confidential.; Achilles; Arrowhead, Autolus; Erytech; Canario Bio, PCI Biotech; OncoQuest; Sobi, Sotio; Trizell;.

I understand that the information above will be published within the journal article, if accepted, and that failure to comply and/or to accurately and completely report the potential financial conflicts of interest could lead to the following: 1) Prior to publication, article rejection, or 2) Post-publication, sanctions ranging from, but not limited to, issuing a correction, reporting the inaccurate information to the authors' institution, banning authors from submitting work to ASN journals for varying lengths of time, and/or retraction of the published work.

Name: Andrew M. Stone
Manuscript ID: K360-2024-000443R1
Manuscript Title: Efficacy and Safety of Nefecon in Patients With Immunoglobulin A Nephropathy From Mainland China: 2-Year NefIgArd Trial Results
Date of Completion: August 12, 2024
Disclosure Updated Date: August 12, 2024

ASN American Society of Nephrology
JASN Journal of the American Society of Nephrology
KIDNEY360 Accessing Our World From Every Angle
CJASN Clinical Journal of the American Society of Nephrology

# ASN Journal Disclosure Form

As per ASN journal policy, I have disclosed any financial relationships or commitments I have held in the past 36 months as included below. I have listed my Current Employer below to indicate there is a relationship requiring disclosure. If no relationship exists, my Current Employer is not listed.

B. Wang reports the following:
Employer: Everest Medicines; and Ownership Interest: Everest Medicines.

I understand that the information above will be published within the journal article, if accepted, and that failure to comply and/or to accurately and completely report the potential financial conflicts of interest could lead to the following: 1) Prior to publication, article rejection, or 2) Post-publication, sanctions ranging from, but not limited to, issuing a correction, reporting the inaccurate information to the authors' institution, banning authors from submitting work to ASN journals for varying lengths of time, and/or retraction of the published work.

Name: Bei Wang
Manuscript ID: K360-2024-000443R1
Manuscript Title: Efficacy and Safety of Nefecon in Patients With Immunoglobulin A Nephropathy From Mainland China: 2-Year NefIgArd Trial Results
Date of Completion: August 2, 2024
Disclosure Updated Date: August 2, 2024

# ASN Journal Disclosure Form

As per ASN journal policy, I have disclosed any financial relationships or commitments I have held in the past 36 months as included below. I have listed my Current Employer below to indicate there is a relationship requiring disclosure. If no relationship exists, my Current Employer is not listed.

L. Ying reports the following:
Employer: Everest Medicines; and Ownership Interest: Everest Medicines, Merck, BMS, etc.

I understand that the information above will be published within the journal article, if accepted, and that failure to comply and/or to accurately and completely report the potential financial conflicts of interest could lead to the following: 1) Prior to publication, article rejection, or 2) Post-publication, sanctions ranging from, but not limited to, issuing a correction, reporting the inaccurate information to the authors' institution, banning authors from submitting work to ASN journals for varying lengths of time, and/or retraction of the published work.

Name: Lisa H. Ying
Manuscript ID: K360-2024-000443R1
Manuscript Title: Efficacy and Safety of Nefecon in Patients With Immunoglobulin A Nephropathy From Mainland China: 2-Year NefIgArd Trial Results
Date of Completion: August 2, 2024
Disclosure Updated Date: August 2, 2024

ASN
American Society of Nephrology
JASN
Journal of the American Society of Nephrology
KIDNEY360
Accessing Our World From Every Angle
CJASN
Clinical Journal of the American Society of Nephrology

# ASN Journal Disclosure Form

As per ASN journal policy, I have disclosed any financial relationships or commitments I have held in the past 36 months as included below. I have listed my Current Employer below to indicate there is a relationship requiring disclosure. If no relationship exists, my Current Employer is not listed.

H. Zhang reports the following:
Employer: Peking University First Hospital; Consultancy: Calliditas; OMEROS; Chinook/Novartis, Ostuka, Roche, Vero, Alexion/AZ; and Advisory or Leadership Role: Calliditas; OMEROS; Chinook/Novartis, Ostuka, Roche, Vero, Alexion/AZ.

I understand that the information above will be published within the journal article, if accepted, and that failure to comply and/or to accurately and completely report the potential financial conflicts of interest could lead to the following: 1) Prior to publication, article rejection, or 2) Post-publication, sanctions ranging from, but not limited to, issuing a correction, reporting the inaccurate information to the authors' institution, banning authors from submitting work to ASN journals for varying lengths of time, and/or retraction of the published work.

Name: Hong Zhang
Manuscript ID: K360-2024-000443R1
Manuscript Title: Efficacy and Safety of Nefecon in Patients With Immunoglobulin A Nephropathy From Mainland China: 2-Year NefIgArd Trial Results
Date of Completion: August 14, 2024
Disclosure Updated Date: June 18, 2024

ASN American Society of Nephrology
JASN Journal of the American Society of Nephrology
KIDNEY360 Accessing Our World From Every Angle
CJASN Clinical Journal of the American Society of Nephrology

# ASN Journal Disclosure Form

As per ASN journal policy, I have disclosed any financial relationships or commitments I have held in the past 36 months as included below. I have listed my Current Employer below to indicate there is a relationship requiring disclosure. If no relationship exists, my Current Employer is not listed.

Z. Zhu reports the following:
Employer: Everest Medicines Limited; Bristol Myers Squibb; and Ownership Interest: Everest Medicines Limited; Bristol Myers Squibb.

I understand that the information above will be published within the journal article, if accepted, and that failure to comply and/or to accurately and completely report the potential financial conflicts of interest could lead to the following: 1) Prior to publication, article rejection, or 2) Post-publication, sanctions ranging from, but not limited to, issuing a correction, reporting the inaccurate information to the authors' institution, banning authors from submitting work to ASN journals for varying lengths of time, and/or retraction of the published work.

Name: Zhengying Zhu
Manuscript ID: K360-2024-000443R1
Manuscript Title: Efficacy and Safety of Nefecon in Patients With Immunoglobulin A Nephropathy From Mainland China: 2-Year NefIgArd Trial Results,
Date of Completion: September 12, 2024
Disclosure Updated Date: September 12, 2024
