# Supplementary material for: Efficacy and Safety of Nefecon in Patients with IgA Nephropathy from Mainland China: 2-Year NefIgArd Trial Results
Source: Kidney360. 2024 Oct 9;5(12):1881–92. doi: 10.34067/KID.0000000583 (PMC11687989; doi:10.34067/KID.0000000583)
Supplement: Supplementary file 2 [file kidney360-5-1881-s002.pdf]

Supplemental material

List of supplemental information

Supplemental statistical methods ... 3

Supplemental Table 1. Sensitivity and supplemental analyses of the time-weighted average of eGFR over 2 years—NefIgArd China cohort and global study population ... 7

Supplemental Table 2. Ratio of eGFR averaged over 12 to 24 months compared with baseline using robust regression—NefIgArd China cohort and global study population (full analysis set) ... 9

Supplemental Table 3. Ratio of time-averaged UPCR or UACR between 12 and 24 months compared with baseline—NefIgArd China cohort and global study population ... 11

Supplemental Table 4. TEAEs in the NefIgArd China cohort (≥15% in the Nefecon treatment group) and global study population (full analysis set) during treatment ... 12

Supplemental Table 5. TEAEs in the NefIgArd China cohort (≥5% in the Nefecon treatment group) and global study population (full analysis set) during follow-up ... 13

Supplemental Table 6. TESAEs in the NefIgArd China cohort during treatment and during follow-up ... 15

Supplemental Figure 1. Patient disposition—NefIgArd China cohort (full analysis set) ... 17

Supplemental Figure 2. Mean absolute and percentage changes in eGFR at 9 and 24 months—NefIgArd China cohort and global study population ... 19

Supplemental Figure 3. Mean percentage change in UPCR at 9 and 24 months—NefIgArd China cohort and global study population ... 20

Supplemental Figure 4. HbA1c shift plots comparing month 3, 6, 9, 12, 18, and 24 with baseline—NefIgArd China cohort ... 21

Confidential - Not for Public Consumption or Distribution

Supplemental Figure 5. Box plots of change from baseline in blood pressure in the NefIgArd China cohort. (A) Systolic blood pressure and (B) diastolic blood pressure 22

Supplemental reference 24

Confidential - Not for Public Consumption or Distribution

Supplemental Statistical Methods

Primary Efficacy End Point Analysis: Time-Weighted Average of eGFR over 2 Years

The estimated glomerular filtration rate (eGFR) data were log-transformed prior to analysis. Data included at baseline and 24 months were the log of the geometric mean of the two replicate values recorded at each time point, respectively. If more than one measurement was recorded within other visit windows, the geometric mean of all measurements within the window was also then used in the analysis. Previous eGFR data from Nefecon trials indicated there could be a small subpopulation of patients with extreme outlying data resulting from very rapid progression of disease. Therefore, robust regression was chosen as the primary analysis method since it down-weights the contribution of outlying data using a predefined algorithm.

In order to handle missing data, the analysis was performed over three phases: an imputation, analysis, and pooling phase, as described below. Missing data could result from the exclusion of data due to rescue medication, the patient having discontinued from the study or, in rare cases, because the patient had died, as well as the lack of recording of data. In all such cases, missing data were imputed conditional on previous outcomes observed within the same patient.

Imputation phase

The first step of the imputation phase was to create data with a monotone data structure having imputed 20 datasets separately within each treatment group. The number of burn-in iterations was set to 200, and observations were sampled every 200 iterations within the same chain for each imputed dataset. An autocorrelation plot was produced to confirm that

Confidential - Not for Public Consumption or Distribution

the separation between samples was long enough to ensure imputations were independent, and a trace plot was also produced to confirm there were no systematic trends.

In the second step of the imputation phase, data were multiply imputed using a regression method sequentially imputing data across successive visits separately by treatment group from each dataset imputed in the first step. This analysis assumes data are missing at random, conditional on previous eGFR recordings.

Missing data were therefore multiply imputed conditional on outcomes previously observed for the same patient and based on the trajectory of outcomes of other patients in the same treatment group, noting that patients in both groups were untreated at the month 12, 18, and 24 time points. Therefore, if a patient's eGFR was deteriorating rapidly at the time of rescue medication or dropout, their imputed values were lower compared with other patients in their treatment group and according to the same overall trajectory of that group.

Analysis Phase

In the analysis phase, the time-weighted average was calculated for each patient within each imputed dataset. Each time point was given the weight stated previously (0.125 for 3, 6, 9, and 12 months and 0.25 for 18 and 24 months). Then, time-weighted average of the log-transformed data was analyzed using robust regression with independent variables of treatment and log-transformed baseline eGFR. M-estimation was used with Huber weights and a cutoff value of 2, with the median method used to estimate the scale parameter. This approach means that standardized residuals with an absolute value of ≤2, corresponding to the central 95% of the data if normally distributed, have equal weight and outlying data are down-weighted according to a prespecified function. Given that dependent variables are categorical and inclusion criteria for the only continuous covariate, log-transformed baseline eGFR, prevents this variable from having outlying values, M-estimation was appropriate.

Confidential - Not for Public Consumption or Distribution

Pooling Phase

In the pooling phase, estimated treatment effects and associated standard errors from each imputation were combined using Rubin's rules to provide an overall treatment effect and associated 95% confidence interval (CI).

Presentation of Results

Results are presented as the ratio of geometric least-squares mean values and the associated 95% CI. This was achieved by exponentiating the treatment effect and 95% CI for the mean difference from baseline in log-transformed values obtained from the robust regression model. To aid interpretation, the treatment effects are expressed as the mean change from baseline in eGFR averaged over 2 years in each treatment group. Mean changes from baseline in eGFR averaged over the 2-year period of treatment and observation were derived directly from the robust regression analysis performed on the log scale by multiplying the baseline geometric mean eGFR, pooled across treatment arms, with the ratio of geometric least-squares means within each arm minus 1. The difference in mean changes from baseline represents the treatment effect expressed as an absolute change. This approach also applies to other analyses of eGFR.

Sensitivity Analysis: Absence of Outliers

A sensitivity analysis was performed using a mixed-effects model for repeated measures including data from all time points over 2 years. The time-weighted average treatment effect was calculated by weighting the treatment effects estimated at each individual time point by 0.125 for 3, 6, 9, and 12 months and 0.25 for 18 and 24 months.

Sensitivity Analysis: Alternative Missing Data Assumptions

An additional sensitivity analysis was performed using different assumptions regarding missing data.

Confidential - Not for Public Consumption or Distribution

In the primary analysis, if a patient discontinued treatment prior to 9 months and did not have subsequent eGFR recordings, their imputed data would be largely based on patients who received 9 months of treatment. This could result in bias if a shorter duration of therapy had a bearing on efficacy and there were a large number of affected patients. In this sensitivity analysis, patients who discontinued early and did not provide further data had data imputed based on the outcomes of other patients who discontinued at the same time but did provide further follow-up data. Missing eGFR values at 18 and 24 months were also imputed conditional on the patient's most recent log-transformed ratio of urine protein–creatinine ratio value compared with baseline (prior to receiving any rescue medication) in case rescue was administered due to a change in urine protein–creatinine ratio value.

Supplemental Analysis: Time-Weighted Average of eGFR over 2 Years—Alternative Handling of Rescue Medication

In this analysis, the Part B primary analysis was repeated with all observed eGFR data included, regardless of the use of rescue medication. This analysis applied a treatment policy estimand and estimated the effect of Nefecon regardless of any other intervention that might have impacted efficacy.

Primary Supportive End Point Analysis: 2-Year eGFR Slope

A primary supportive analysis of the 2-year eGFR slope was performed using a random coefficients approach. The analysis was performed using all data recorded up until the 24-month visit, with data impacted by rescue medication excluded. In this analysis, the actual time measurements taken were used as the time variable, and in particular, repeat month-24 measurements were included as separate observations.

In this analysis, after exclusion of data impacted by rescue medication, no missing data were imputed, with the 2-year slope analysis based solely on observed data.

Confidential - Not for Public Consumption or Distribution

Supplemental Table 1. Sensitivity and supplemental analyses of the time-weighted average of eGFR over 2 years—NefIgArd China cohort and global study population

Analysis	China Cohort				Global Study Population			
	N	Nefecon 16 mg/d vs Placebo Treatment Effect in Average eGFR Difference over 2 Years (95% CI), ml/min per 1.73 m^2	Mean Change from Baseline in eGFR Averaged over 2 Years (95% CI), ml/min per 1.73 m^2		N	Nefecon 16 mg/d vs Placebo Treatment Effect in Average eGFR Difference over 2 Years (95% CI), ml/min per 1.73 m^2	Mean Change from Baseline in eGFR Averaged over 2 Years (95% CI), ml/min per 1.73 m^2	
			Nefecon 16 mg	Placebo			Nefecon 16 mg	Placebo
Primary robust regression analysis (FAS)	62	9.6 (2.0 to 19.8)	−3.7 (−8.9 to 2.0) *n*=32	−13.3 (−18.1 to −8.0) *n*=30	364	5.1 (3.2 to 7.4)	−2.5 (−3.9 to −1.0) *n*=182	−7.5 (−8.8 to −6.2) *n*=182
1) Robust regression analysis (per-protocol analysis set)	58	8.3 (0.9 to 18.0)	−3.6 (−8.7 to 2.1) *n*=31	−11.9 (−16.8 to −6.5) *n*=27	325	5.2 (3.4 to 7.6)	−2.0 (−3.4 to −0.6) *n*=161	−7.2 (−8.5 to −5.9) *n*=164
2) MMRM analysis in absence of outliers (FAS)	62	11.8 (2.9 to 24.2)	−3.9 (−10.5 to 3.5) *n*=32	−15.7 (−20.9 to −9.8) *n*=30	358	5.2 (3.1 to 7.8)	−3.0 (−4.6 to −1.4) *n*=179	−8.2 (−9.6 to −6.7) *n*=179
3) Robust regression analysis with alternative	62	9.3 (2.0 to 19.1)	−3.8 (−8.7 to 1.6) *n*=32	−13.2 (−17.9 to −7.9) *n*=30	364	5.1 (3.2 to 7.6)	−2.6 (−4.0 to −1.1) *n*=182	−7.7 (−9.1 to −6.3) *n*=182

Confidential - Not for Public Consumption or Distribution

missing data assumptions[a] (FAS)								
4) Robust regression analysis including data observed after receiving rescue medication (FAS)	62	6.7 (0.3 to 14.9)	−4.5 (−9.1 to 0.5) *n*=32	−11.3 (−15.3 to −6.8) *n*=30	364	4.8 (2.9 to 7.2)	−2.8 (−4.2 to −1.3) *n*=182	−7.6 (−8.9 to −6.2) *n*=182

"*n*" represents the number of patients included in the analysis. In all robust regression analyses, missing data were multiply imputed prior to analysis. In the MMRM analysis, missing data were implicitly imputed for those patients without a valid observed result. "*N*" represents the total number of patients included who either had data observed or imputed.

eGFR was calculated by the central laboratory using the CKD-EPI formula.

[a]This analysis uses alternative missing data assumptions for patients who discontinued from treatment early and did not provide complete eGFR data. In this analysis, missing data are imputed differently to the primary analysis depending on (1) duration of treatment received, and (2) data from 12 months onward are imputed conditional on their most recent log-transformed ratio of UPCR value compared with baseline (prior to receiving any rescue medication).

CI, confidence interval; CKD-EPI, Chronic Kidney Disease Epidemiology Collaboration; eGFR, estimated glomerular filtration rate; FAS, full analysis set; MMRM, mixed-effects model for repeated measures; UPCR, urine protein–creatinine ratio.

Confidential - Not for Public Consumption or Distribution

Supplemental Table 2. Ratio of eGFR averaged over 12 to 24 months compared with baseline using robust regression—NefIgArd China cohort and global study population (full analysis set)

Analysis	China Cohort		Global Study Population	
	Nefecon 16 mg/d (*N*=32)	Placebo (*N*=30)	Nefecon 16 mg/d (*N*=182)	Placebo (*N*=182)
Ratio of geometric LS mean eGFR averaged over 12 to 24 months compared with baseline (95% CI)	0.90 (0.79 to 1.01)	0.72 (0.63 to 0.83)	0.93 (0.90 to 0.96)	0.84 (0.81 to 0.86)
Mean change from baseline in eGFR averaged over 12 to 24 months (95% CI), ml/min per 1.73 m^2	−6.1 (−12.2 to 0.7)	−16.4 (−21.9 to −10.2)	−4.1 (−5.7 to −2.4)	−9.1 (−10.6 to −7.6)
Comparison of Nefecon 16 mg/d vs placebo:				
Ratio of geometric LS means (95% CI)	1.24 (1.03 to 1.50)		1.11 (1.06 to 1.16)	
Mean difference in eGFR averaged over 12 to 24 months (95% CI), ml/min per 1.73 m^2	10.3 (1.4 to 22.6)		5.0 (2.9 to 7.7)	

All patients in the Part B full analysis set were included in the analysis, which implicitly imputed missing data for those patients without a valid eGFR result at the respective time point.

The mean change in eGFR was also derived directly from the robust regression model.

eGFR was calculated by the central laboratory using the CKD-EPI formula.

Confidential - Not for Public Consumption or Distribution

CI, confidence interval; CKD-EPI, Chronic Kidney Disease Epidemiology Collaboration; eGFR, estimated glomerular filtration rate; LS, least-squares.

Confidential - Not for Public Consumption or Distribution

Supplemental Table 3. Ratio of time-averaged UPCR or UACR between 12 and 24 months compared with baseline—NefIgArd China cohort and global study population

Proteinuria Measurement	China Cohort			Global Study Population		
	Percentage Change from Baseline		Percentage Reduction vs Placebo (95% CI)[a]	Percentage Change from Baseline		Percentage Reduction vs Placebo (95% CI)[a]
	Nefecon 16 mg/d (95% CI), *N*=31	Placebo (95% CI), *N*=30		Nefecon 16 mg/d (95% CI), *N*=172	Placebo (95% CI), *N*=173	
UPCR	−42 (−57 to −22)	20 (−11 to 61)	52 (28 to 68)	−40 (−46 to −34)	1 (−9 to 12)	41 (32 to 49)
UACR	−50 (−63 to −34)	5 (−22 to 42)	53 (28 to 69)	−48 (−54 to −42)	−4 (−15 to 8)	46 (37 to 55)

[a]The percentage difference between Nefecon and placebo was calculated as 100 × (the ratio of the ratio of mean / baseline in the Nefecon group and the ratio of mean / baseline in the placebo group - 1). By contrast, the percentage change in each treatment group was calculated as 100 × (the ratio of mean / baseline in each treatment group - 1).

CI, confidence interval; UACR, urine albumin–creatinine ratio; UPCR, urine protein−creatinine ratio.

Confidential - Not for Public Consumption or Distribution

Supplemental Table 4. TEAEs in the NefIgArd China cohort (≥15% in the Nefecon treatment group) and global study population (full analysis set) during treatment

Preferred Term<br><br>Patients, *n* (%)	China Cohort		Global Study Population	
	**Nefecon 16 mg (*N*=32)**	**Placebo (*N*=30)**	**Nefecon 16 mg (*N*=182)**	**Placebo (*N*=182)**
Patients with any TEAE	31 (96.9)	24 (80.0)	159 (87.4)	125 (68.7)
Upper respiratory tract infection	10 (31.3)	3 (10.0)	10 (5.5)	10 (5.5)
Edema peripheral[a]	6 (18.8)	3 (10.0)	31 (17.0)	7 (3.8)
Menstrual disorder	6 (18.8)	1 (3.3)	6 (3.3)	1 (0.5)
Neutrophil count increased	6 (18.8)	0 (0.0)	5 (2.7)	0 (0.0)
White blood cell count increased	5 (15.6)	0 (0.0)	8 (4.4)	0 (0.0)

% = 100 × *n*/*N*.

TEAEs were defined as AEs that occurred for the first time after dosing with study treatment or existed before but worsened in severity or relationship to study treatment after dosing. AEs that started >14 days after the last dose of study treatment were excluded from the summary. The last dose was defined as the last dose the patient received, including the tapering period, regardless of the duration of treatment.

AE reported terms were coded using the Medical Dictionary for Regulatory Activities Version 22.0.

[a]Preferred terms were grouped for edema peripheral (edema peripheral and peripheral swelling).

AE, adverse event; TEAE, treatment emergent adverse event.

Confidential - Not for Public Consumption or Distribution

Supplemental Table 5. TEAEs in the NefIgArd China cohort (≥5% in the Nefecon treatment group) and global study population (full analysis set) during follow-up

Preferred Term	China Cohort		Global Study Population[S1]	
Patients, *n* (%)	Nefecon 16 mg (*N*=32)	Placebo (*N*=30)	Nefecon 16 mg (*N*=182)	Placebo (*N*=182)
Number of patients who had a study visit during the follow-up period, *N*	32	30	175	174
Patients with any TEAE starting >14 days after last dose	23 (71.9)	25 (83.3)	127 (73)	124 (71)
Coronavirus infection	12 (37.5)	11 (36.7)	26 (15)	30 (17)
Pyrexia	3 (9.4)	5 (16.7)	<5%	<5%
Dermatitis[a]	2 (6.3)	0 (0.0)	<5%	<5%
Hyperuricemia	2 (6.3)	3 (10.0)	<5%	<5%
Upper respiratory tract infection	2 (6.3)	3 (10.0)	<5%	<5%
Cough	2 (6.3)	1 (3.3)	<5%	<5%
Diarrhea	2 (6.3)	1 (3.3)	<5%	<5%
Pulmonary mass	2 (6.3)	1 (3.3)	NR	NR
Hepatic steatosis	2 (6.3)	0 (0.0)	NR	NR
Edema peripheral[a]	0 (0.0)	1 (3.3)	14 (8)	10 (6)
Gout	NR	NR	11 (6)	8 (5)
Hypertension	0 (0.0)	5 (16.7)	10 (6)	12 (7)

% = 100 × *n*/*N*.

Data from the global study population are rounded to the nearest whole percentage to align with the published source material.[S1]

TEAEs were defined as AEs that occurred for the first time after dosing with study treatment or existed before but worsened in severity or relationship to study treatment after dosing.

Confidential - Not for Public Consumption or Distribution

An AE that occurred >14 days after the last dose of study treatment was not considered a TEAE if it was not the first occurrence, did not worsen in severity compared with the previous occurrence, and/or was not related to study treatment. The last dose was defined as the last dose the patient received, including the tapering period, regardless of the duration of treatment. AE reported terms were coded using the Medical Dictionary for Regulatory Activities Version 22.0.

[a]Preferred terms were grouped for dermatitis (dermatitis, hand dermatitis, perioral dermatitis, seborrheic dermatitis, and eczema) and edema peripheral (edema peripheral and peripheral swelling).

AE, adverse event; NR, not reported; TEAE, treatment emergent adverse event.

Confidential - Not for Public Consumption or Distribution

Supplemental Table 6. TESAEs in the NefIgArd China cohort during treatment and during follow-up

Patient	AE Preferred Term	Days After Start of Treatment	Duration of AE (Days)	Severity	Assessed as Related to Study Treatment by Investigator
**Treatment phase: Nefecon 16 mg**					
#1	Hypertension	196	44	Moderate	Yes
**Follow-up phase: Nefecon 16 mg**					
#2	End stage kidney disease	569	Ongoing	Severe	No
#3	Upper respiratory tract infection	321	5	Mild	Yes
**Follow-up phase: Placebo**					
#4	Large intestine polyp	518	2	Moderate	No
#5	Acute kidney injury	387	45	Mild	No
	Atrioventricular block complete	505	38	Moderate	No
	Renal failure	727	Ongoing	Severe	No
#6	Chronic kidney disease	471	Ongoing	Moderate	No
#7	Cardiac failure	475	197	Moderate	No
	Hypothyroidism	481	247	Moderate	No
#8	End stage renal disease	527	Ongoing	Moderate	No
#9	Bile duct stone	424	27	Moderate	No
#10	End stage renal disease	509	Ongoing	Moderate	No
	Cardiac failure acute	614	7	Severe	No

Confidential - Not for Public Consumption or Distribution

TESAEs were defined as SAEs that occurred for the first time after dosing with study treatment or existed before but worsened in severity or relationship to study treatment after dosing. An SAE that occurred >14 days after the last dose of study treatment was not considered a TESAE if it was not the first occurrence, did not worsen in severity compared with the previous occurrence, and/or was not related to study treatment.

The last dose was defined as the last dose the patient received, including the Tapering Period, regardless of the duration of treatment.

AE reported terms were coded using the Medical Dictionary for Regulatory Activities Version 22.0.

Duration = End Date – Start Date + 1.

AE, adverse event; SAE, serious adverse event; TESAE, treatment-emergent serious adverse event.

Confidential - Not for Public Consumption or Distribution

Supplemental Figure 1. Patient disposition—NefIgArd China cohort (full analysis set)

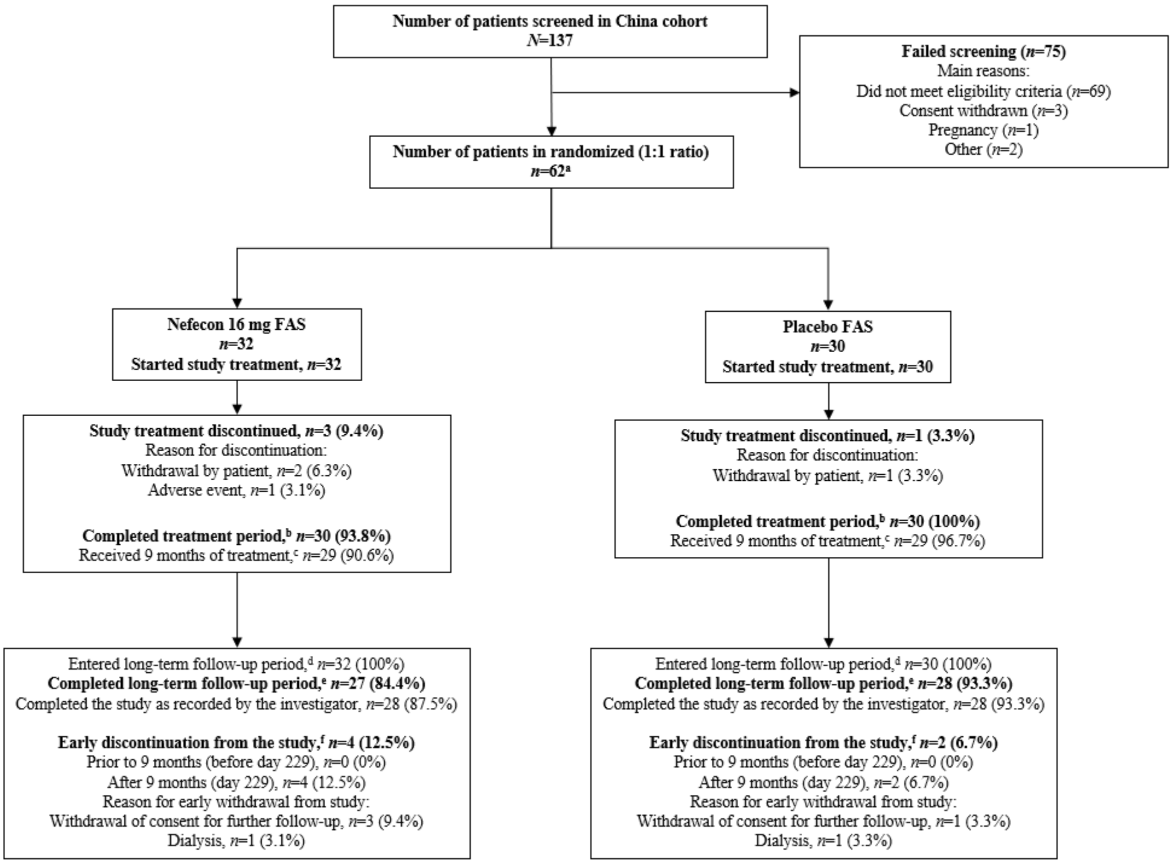

[a]Of the 62 patients randomized, 33 patients in China were randomized during the global study, with a further 29 patients randomized after global recruitment had ended.

[b]"Completed treatment period" was defined as the patient having at least one valid urine protein–creatinine ratio value available in the 9-month visit window (day 229 to day 319).

[c]The patient was considered to have received 9 months of treatment if the date of last dose (excluding doses received in the tapering period) – date of first dose + 1 ≥255.

[d]The patient was defined as having entered the "long-term follow-up period" if they attended at least one study visit or had any adverse event recorded that was more than 14 days after the last dose of study treatment (including tapering).

[e]"Completed long-term follow-up period" was defined as the patient having at least one valid eGFR value within the 24-month visit window (day 640 to day 821).

Confidential - Not for Public Consumption or Distribution

[f]As recorded by the investigator.

eGFR, estimated glomerular filtration rate; FAS, full analysis set.

Confidential - Not for Public Consumption or Distribution

Supplemental Figure 2. Mean absolute and percentage changes in eGFR at 9 and 24 months—NefIgArd China cohort and global study population

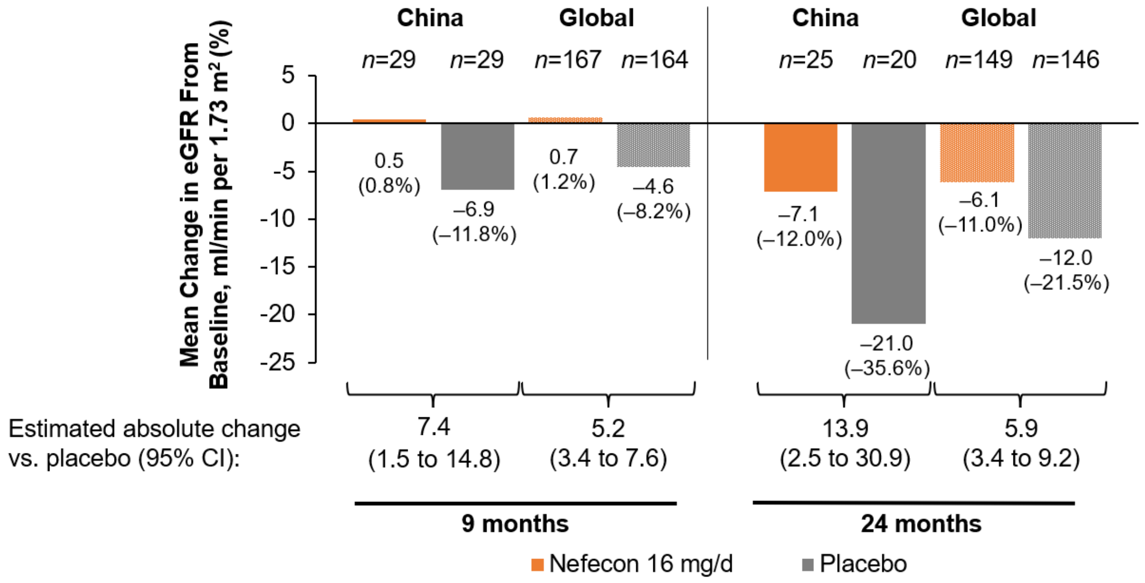

CI, confidence interval (profile-likelihood method); eGFR, estimated glomerular filtration rate.

Confidential - Not for Public Consumption or Distribution

Supplemental Figure 3. Mean percentage change in UPCR at 9 and 24 months—NefIgArd China cohort and global study population

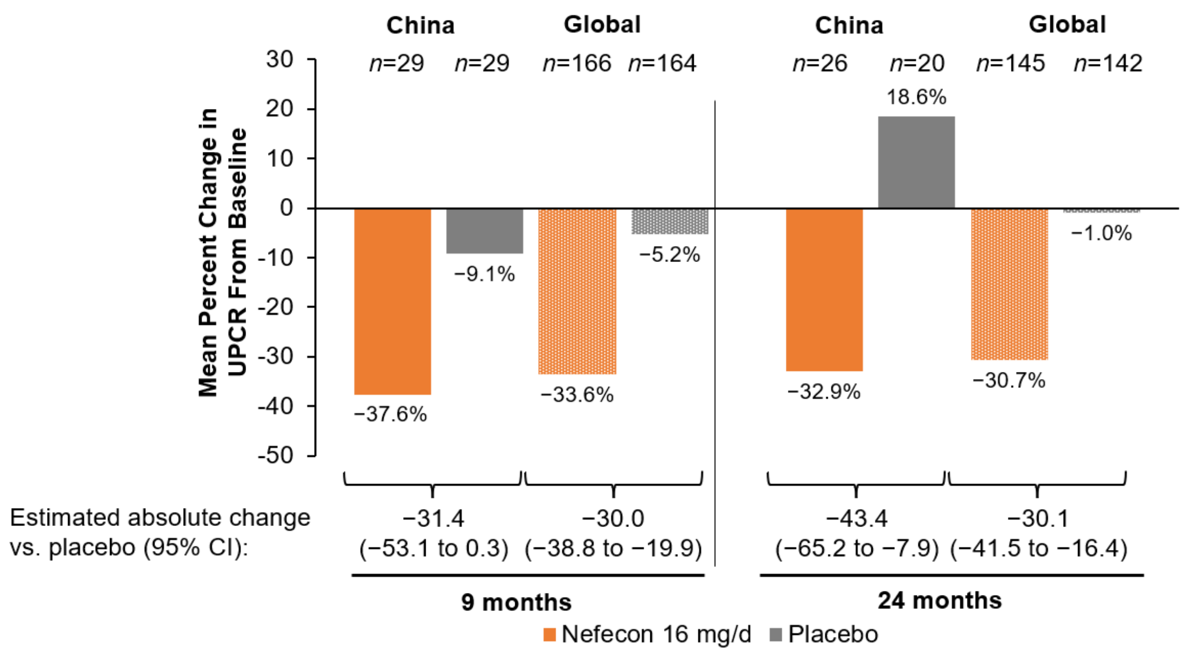

CI, confidence interval; UPCR, urine protein–creatinine ratio.

Confidential - Not for Public Consumption or Distribution

Supplemental Figure 4. HbA1c shift plots comparing month 3, 6, 9, 12, 18, and 24 with baseline—NefIgArd China cohort

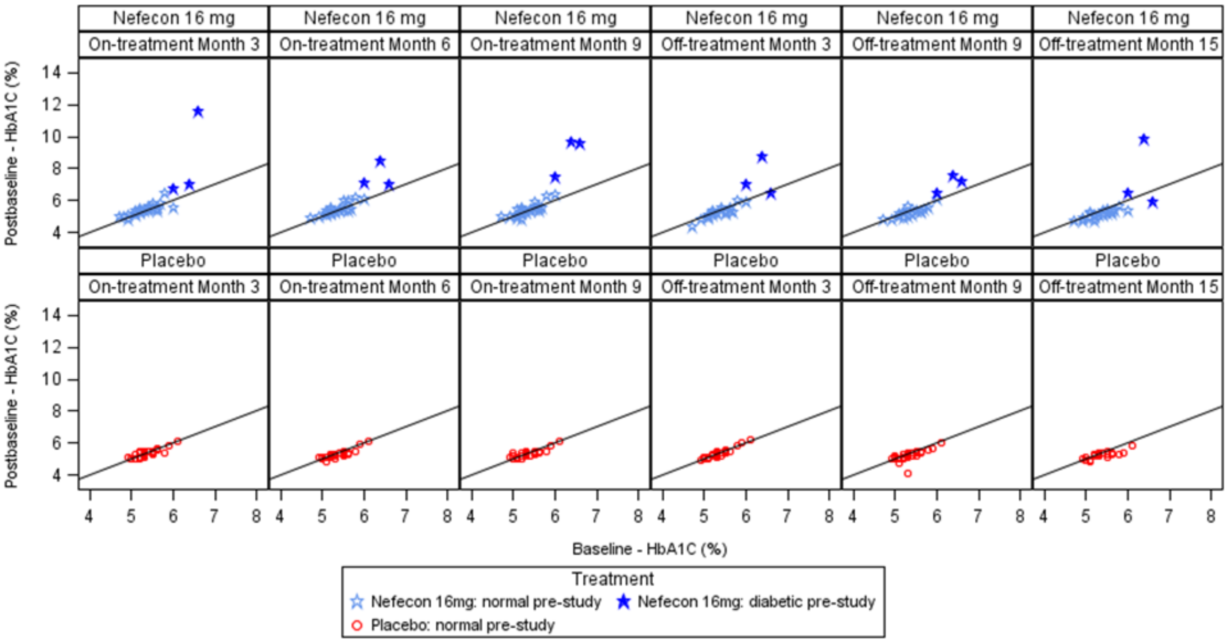

HbA1c, hemoglobin A1c.

Confidential - Not for Public Consumption or Distribution

Supplemental Figure 5. Box plots of change from baseline in blood pressure in the NefIgArd China cohort. (A) Systolic blood pressure and (B) diastolic blood pressure

A.

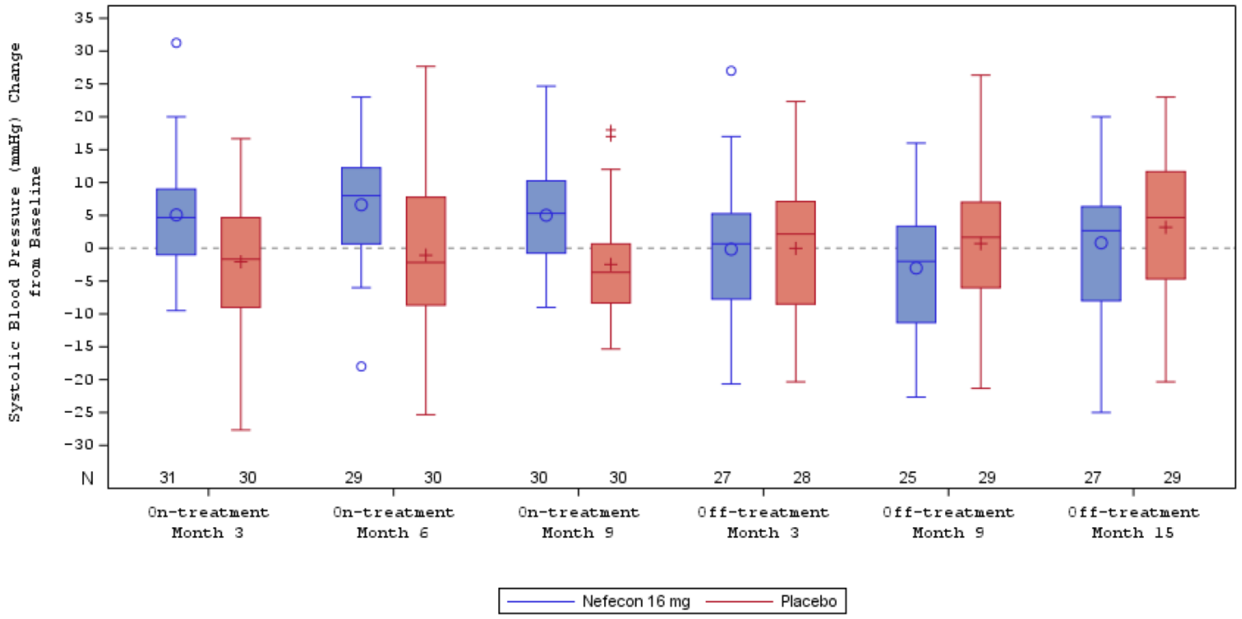

B.

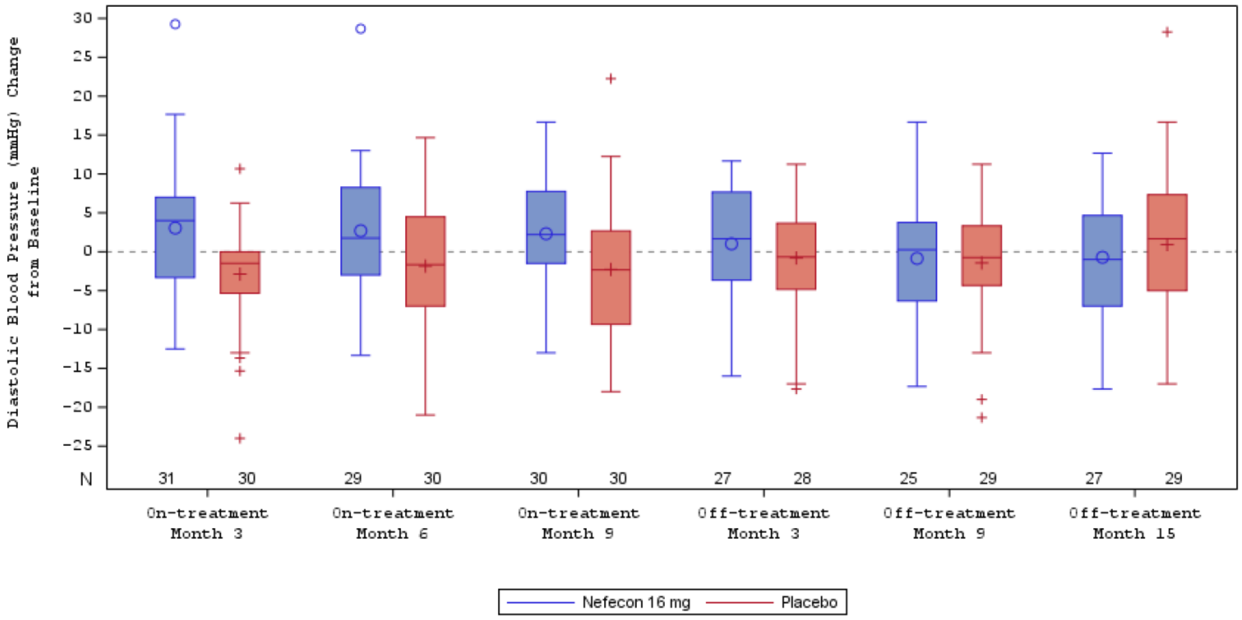

Confidential - Not for Public Consumption or Distribution

Boxes show quartile 1, median, quartile 3, and whiskers extend to the most extreme point less than or equal to 1.5 interquartile ranges. Outliers are included in calculations.

Confidential - Not for Public Consumption or Distribution

Supplemental reference

S1. Lafayette R, Kristensen J, Stone A, Floege J, Tesar V, Trimarchi H, Zhang H, Eren N, Paliege A, Reich HN, Rovin BH, Barratt J, NefIgArd trial Investigators: Efficacy and safety of a targeted-release formulation of budesonide in patients with primary IgA nephropathy (NefIgArd): 2-year results from a randomised phase 3 trial. *Lancet* 402: 859–870, 2023. doi:10.1016/S0140-6736(23)01554-4

Confidential - Not for Public Consumption or Distribution
